# Modulation of S100β and Inflammatory Signalling by Isorhamnetin Enhances Peripheral Nerve Regeneration

**DOI:** 10.3390/ijms27083624

**Published:** 2026-04-18

**Authors:** Ammara Tehreem, Arslan Iftikhar, Ikram Ullah Khan, Ghulam Hussain

**Affiliations:** 1 Neuroregeneration and Genetics Laboratory (NGL), Department of Physiology, Faculty of Life Sciences, Government College University, Faisalabad 38000, Pakistan; ammara.tehreem4@gmail.com (A.T.); arslaniftikhar@gcuf.edu.pk (A.I.); 2 Department of Pharmaceutics, Faculty of Pharmaceutical Sciences, Government College University, Faisalabad 38000, Pakistan; ikramglt@gmail.com

**Keywords:** isorhamnetin, peripheral nerve injury, MDA, oxidative stress, neuroprotection

## Abstract

Peripheral nerve injury is a leading cause of disability, which can result in partial or complete loss of motor, sensory, and autonomic function, and currently, there is no effective treatment for this incapacitating condition. It is important to identify new compounds that enable rapid and complete functional recovery. This study evaluated the effects of isorhamnetin (ISO) on functional rehabilitation in a mouse model of sciatic nerve injury. A total of 30 BALB/c mice, aged 8–10 weeks, were randomly assigned to three groups: sham, control, and treatment (*n* = 10/group). The mice in the ISO and Ctrl groups were operated on, whilst the animals in the sham group had their sciatic nerves exposed but left intact without crushing. The Ctrl and Sham groups received DMSO and normal saline intraperitoneally in equal volumes. In contrast, the ISO-treated group received ISO (10 mg/kg) dissolved in DMSO intraperitoneally from the day of nerve crush until the end of the study. All groups were fed regular chow and provided with sufficient water throughout the experiment. Behavioural analyses evaluated sensorimotor function recovery. Biochemical and haematological assays quantified oxidative stress markers and total blood count, while morphometric analysis determined structural recovery of muscle fibers. Nerve regeneration was indirectly evaluated by analyzing S100β protein levels and proinflammatory cytokines (IL-6 and TNF-α) expression. In the mouse model, ISO treatment resulted in substantial improvement in sensorimotor function recovery (*p* < 0.001). A substantial difference (*p* < 0.001) in blood glucose levels and oxidative stress markers was observed among all groups. The treated group displayed a remarkable improvement in the cross-sectional area of muscle fibers. At the end of the study, it was noted that ISO treatment significantly downregulated the expression of S100β, TNF-α, and IL-6, suggesting a positive impact of ISO on nerve regeneration. These findings indicate that ISO expedites the restoration of sensorimotor function following sciatic nerve injury by modulating S100β and proinflammatory cytokine expression and improving oxidative stress.

## 1. Introduction

Peripheral nerve injury (PNI) is a prevalent clinical issue that can result from a variety of situations, including sharp objects, firearms, stretching, compression, ischemia, and iatrogenic injury [1,2]. PNIs lead to numbness, reduced motor and sensory abilities, neuropathic pain, or even paralysis [3]. PNI was estimated to affect 13 to 23 people out of 100,000 annually; the effects might result in permanent impairment and a lifetime condition [4]. The degree of nerve damage and the likelihood of healing depend upon the nature and severity of the injury. The pathophysiology of PNI is significantly influenced by oxidative stress and inflammation [5]. Following PNI, a rapid proinflammatory reaction triggers the removal of tissue debris (Wallerian degeneration), facilitating successful regeneration [6]. As research has advanced, it has become increasingly evident that inflammation plays a role in nerve damage healing and regeneration. Excessive inflammation impairs nerve function recovery and, in extreme cases, may lead to neurological disorders [7]. On the other hand, oxidative stress causes alterations in nerve structure, including axonal degeneration, segmental demyelination, and Schwann cell death [8,9].

Axonal outgrowth is an intricate process that requires the coordinated action of several cell types, including macrophages, which aid in the removal of debris and create an environment conducive to nerve regeneration; Schwann cells, which support and guide the developing nerve fibers; and neurons, all of which are essential for the complete regeneration of damaged nerves. This process can occur over weeks to months [10,11]. The complete functional recovery remains a problem, even with management strategies [12]. In this context, phytochemicals are attracting attention for their therapeutic potential [13].

Structurally, polyphenols are characterized by the presence of one or more hydroxyl (-OH) groups attached to aromatic rings, and the molecular structure of these compounds results in high absorption in their free form because of their lipophilic characteristics [14]. Their neuroprotective, anti-inflammatory, and antioxidant qualities make them attractive options for both preventing and treating neurological diseases [15]. They are known to stimulate and regulate the regenerative process after tissue injury due to their antioxidant and anti-inflammatory properties [16,17]. Polyphenols consist of four groups: lignans, stilbenes, flavonoids, and phenolic acids, depending on the number of phenol rings and the structural elements that keep the rings together [18].

The Mediterranean diet has the highest concentration of flavonoids. Since many are yellow, the term “flavonoid” derives from the Latin word for “yellow” [19]. Flavonoids possess neuroprotective properties that improve cognitive function and lower neuroinflammation in neurodegenerative diseases [20]. Quercetin is one of the most potent plant antioxidants. It belongs to the family of polyphenols known as flavonols, which is a subclass of flavonoids [21].

ISO, a naturally occurring flavonoid found in many plants and plant-based diets, is a direct metabolite of quercetin [22]. It is used to treat cardiovascular, neurological, and renal problems due to its anti-inflammatory and antioxidant qualities [23,24]. ISO is a helpful hypoglycemic medication for the treatment of type 2 diabetes due to its multifaceted effects, including lowering insulin resistance, enhancing skeletal muscle glucose uptake, improving lipid profiles, and reducing inflammation and oxidative stress [25]. In mice with type 2 diabetes, ISO can improve dyslipidemia by reducing the production of inflammatory cytokines [26]. It may contribute to protection against cerebral vascular degeneration caused by ischemia, as it protects human brain microvascular endothelial cells from cytotoxicity induced by oxygen-glucose deprivation [27]. Another study suggested that ISO may help prevent neurodegenerative disorders such as Alzheimer’s disease [23], thereby supporting its potential neuroprotective properties. Translational research using animal models of nerve regeneration has the potential to advance therapeutic approaches in veterinary and human health [28,29]. The present study investigates the effect of ISO on the rate of sensorimotor function restoration in an experimentally induced mouse model of sciatic nerve injury.

## 2. Results

### 2.1. Effects of ISO on the Viability of HepG2 Cells

**Figure 1:**
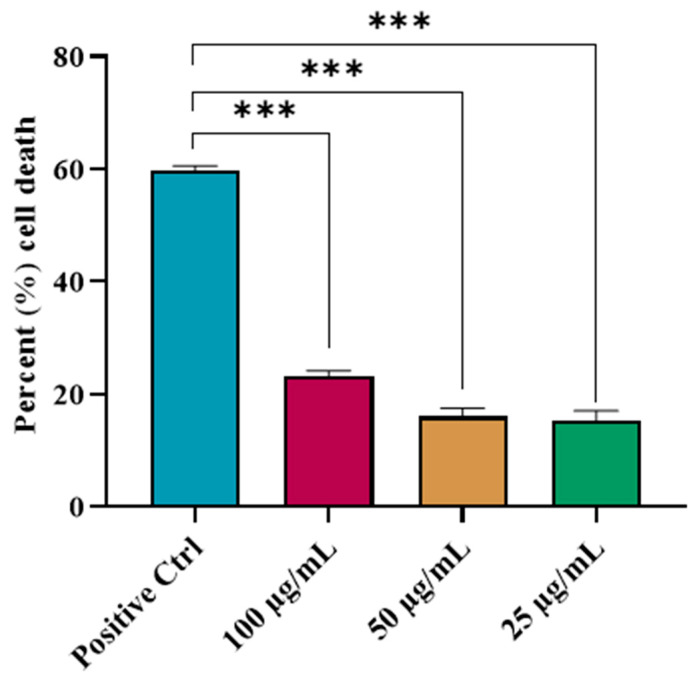
Effects of ISO on the viability of HepG2 cells: One-way ANOVA revealed a significant difference (*** *p* < 0.001) in the effect of ISO on the viability of HepG2 cells when compared with the positive control (doxorubicin-treated group). ISO = Isorhamnetin.

It was noted that the ISO treatment resulted in a reduction in the HepG2 cell viability in a dose-dependent manner. However, it yielded significantly (*** *p* < 0.001) less HepG2 cell death when compared with the positive control (doxorubicin-treated) group (Figure 1). ISO demonstrated considerably lower toxicity than doxorubicin, indicating a less pronounced cytotoxic profile. This may suggest that ISO is relatively safer while still exerting biological activity.

### 2.2. Effect of ISO Treatment on Body Mass and Food Intake

**Figure 2:**
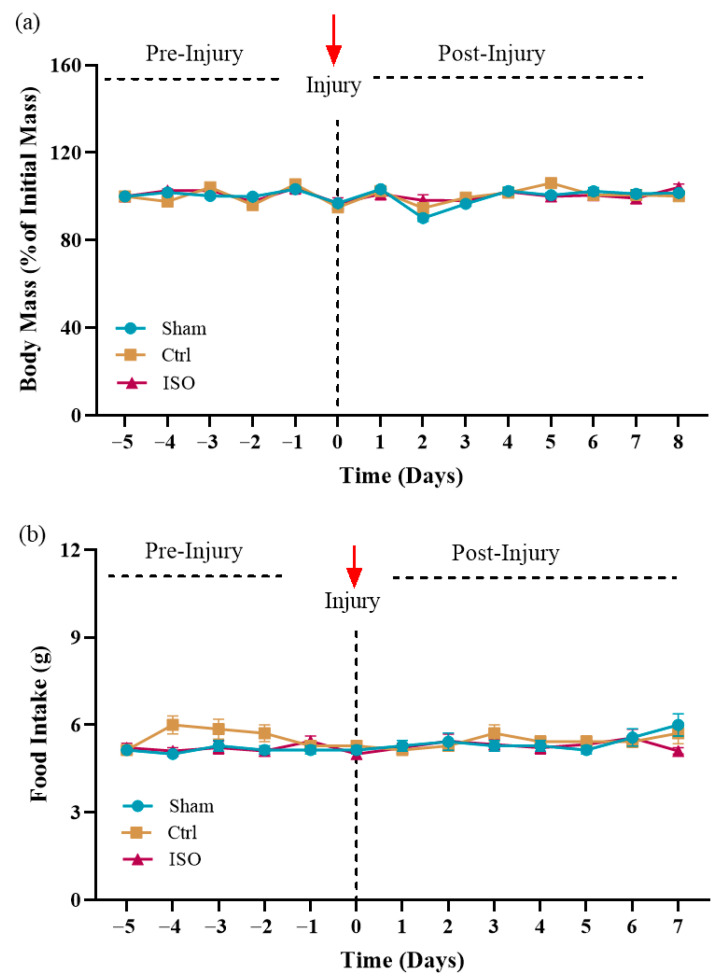
Effects of ISO treatment on body mass and food intake: (**a**) Body mass presented a non-significant effect (*p* = 0.060); (**b**) Food intake showed no significant difference (*p* = 0.144) following treatment. Ctrl = control, ISO = Isorhamnetin.

Food intake and body mass were monitored throughout the experimental period. No significant differences were observed in body mass (*p* = 0.060) or dietary intake (*p* = 0.144) among the Sham, Ctrl, and ISO-treated groups (Figure 2a,b).

### 2.3. ISO Facilitates Motor Function Restoration

**Figure 3:**
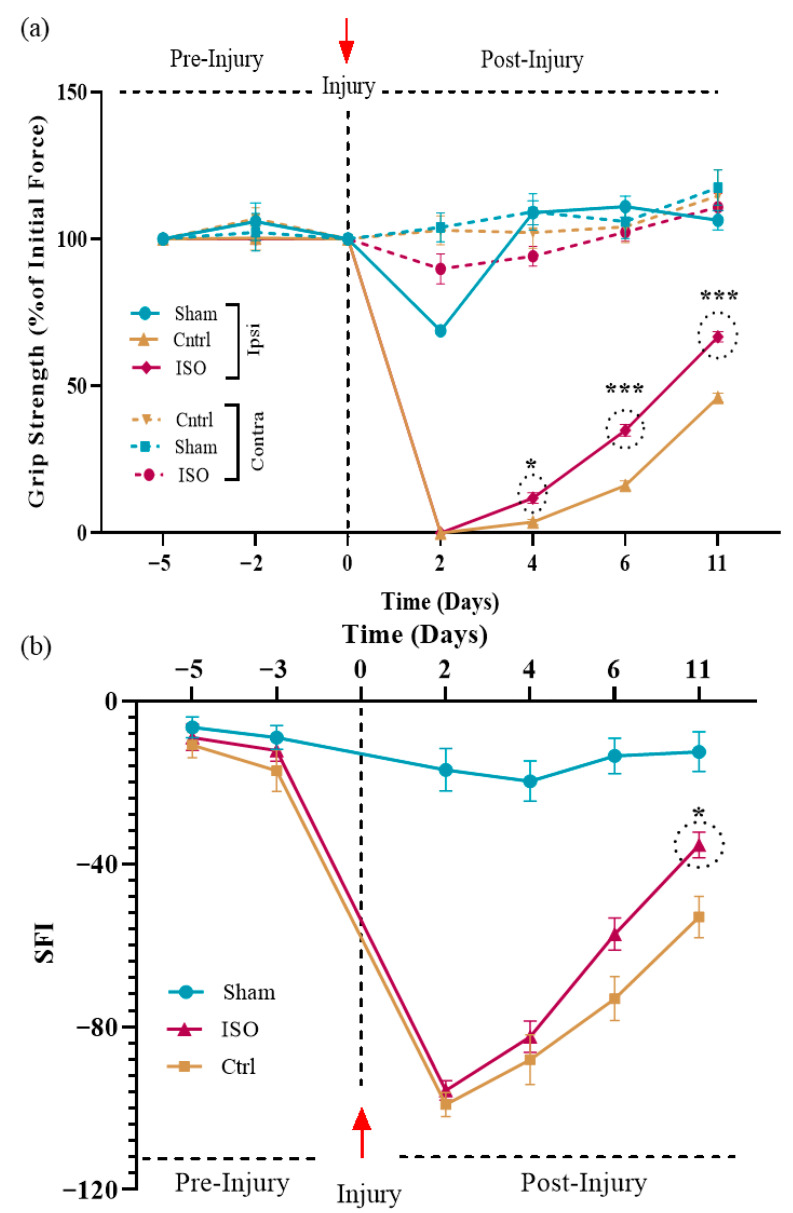
Effect of ISO treatment on motor function recovery. (**a**) Muscle grip strength was significantly improved in the ISO-treated group (* *p* < 0.05, *** *p* < 0.001) compared with the Ctrl and Sham groups. (**b**) The SFI indicated an improved walking pattern in the ISO-treated animals, and this difference was statistically significant (* *p* < 0.05). Ctrl = control, ISO = Isorhamnetin.

Motor function was assessed using SFI and grip strength measurements. The ISO-treated group exhibited significantly improved motor performance compared with the control group. Muscular grip strength and SFI values showed a significant difference among the ISO-treated, Ctrl, and Sham groups (*** *p* < 0.001) (Figure 3a,b).

### 2.4. ISO Improves Sensory Functional Recovery

**Figure 4:**
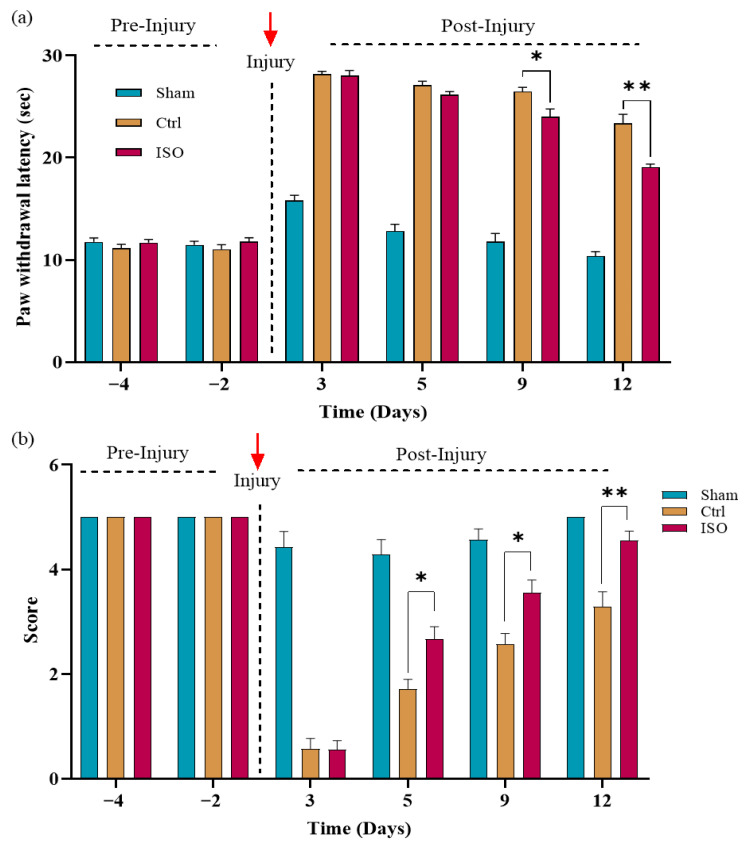
Effect of ISO treatment on sensory function restoration. (**a**) The paw withdrawal latency revealed significant differences (* *p* < 0.05, ** *p* < 0.01). (**b**) The pinprick score showed a significant difference among the Ctrl, sham, and ISO-treated groups (* *p* < 0.05, ** *p* < 0.01). Ctrl = control, ISO = Isorhamnetin.

Sensory functions were evaluated using the Hot-plate and Pinprick tests. The ISO-treated group exhibited significant (* *p* < 0.05, *** *p* < 0.001) improvement in sensory function recovery compared with the Ctrl and Sham groups (Figure 4a,b).

### 2.5. Effect of ISO on Random Blood Glucose Levels

**Figure 5:**
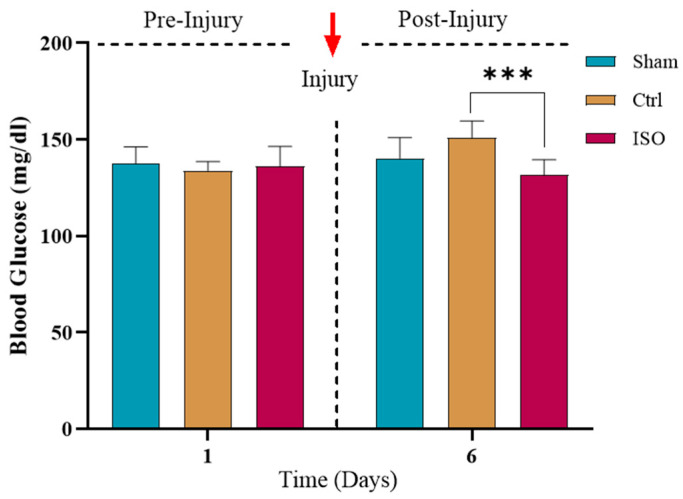
Effect of ISO treatment on blood glucose levels. The ISO-treated group showed a significant decrease in glucose levels (*** *p* < 0.001) compared to the Ctrl group after treatment. Ctrl = control, ISO = Isorhamnetin.

A statistically significant (*** *p* < 0.001) reduction in blood glucose levels was observed in the ISO-treated group compared with the Ctrl group (Figure 5).

### 2.6. ISO Modulates Oxidative Stress Markers

**Figure 6:**
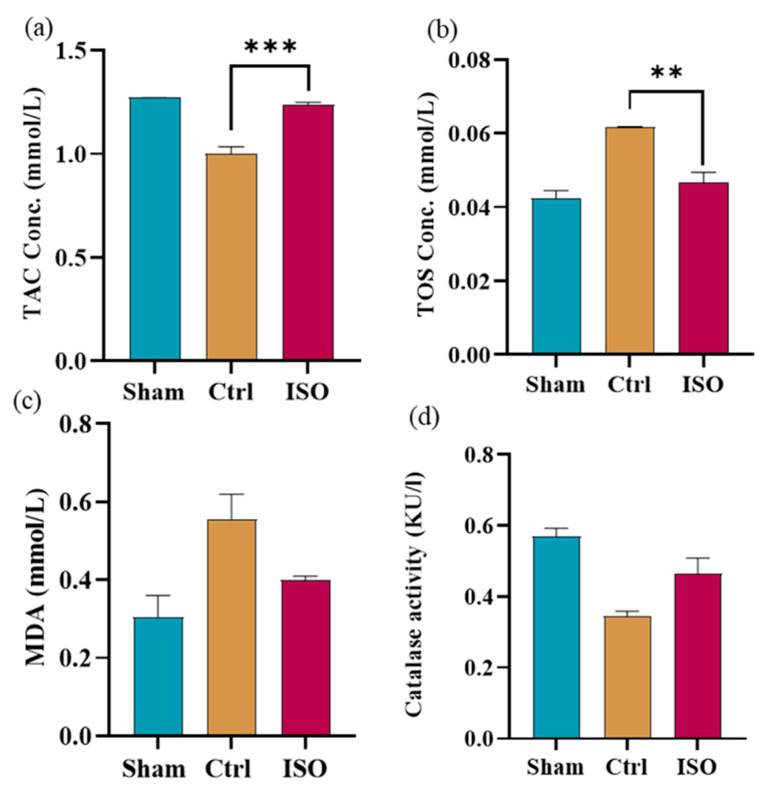
Effect of ISO on TAC, TOS, MDA, and Catalase activity. A one-way ANOVA with multiple comparisons revealed that ISO had greater antioxidant potential than Ctrl. (**a**) ISO-treated group significantly amplified TAC (*** *p* < 0.001), and (**b**) declined levels of TOS (** *p* = 0.003) when compared to the Ctrl group. (**c**) The effect of ISO treatment on MDA was not significant (*p* = 0.181), with values lower than those in the Ctrl group. (**d**) The ISO-treated group showed a significantly elevated Catalase activity (*p* = 0.032) compared with the Ctrl group. Ctrl = Control, ISO = Isorhamnetin.

ISO administration was associated with a reduced oxidative stress as assessed in serum samples collected after the experiment. A statistically significant difference (*** *p* < 0.001) in TAC and TOS levels was observed between the ISO-treated and Ctrl groups (Figure 6a,b). Although no significant difference in MDA levels was observed (*p* = 0.081), it was lower in the treated group, and a statistically significant ( *p* = 0.032) difference in Catalase activity was observed among all groups (Figure 6c,d).

### 2.7. ISO Regulates S100β Expression

**Figure 7:**
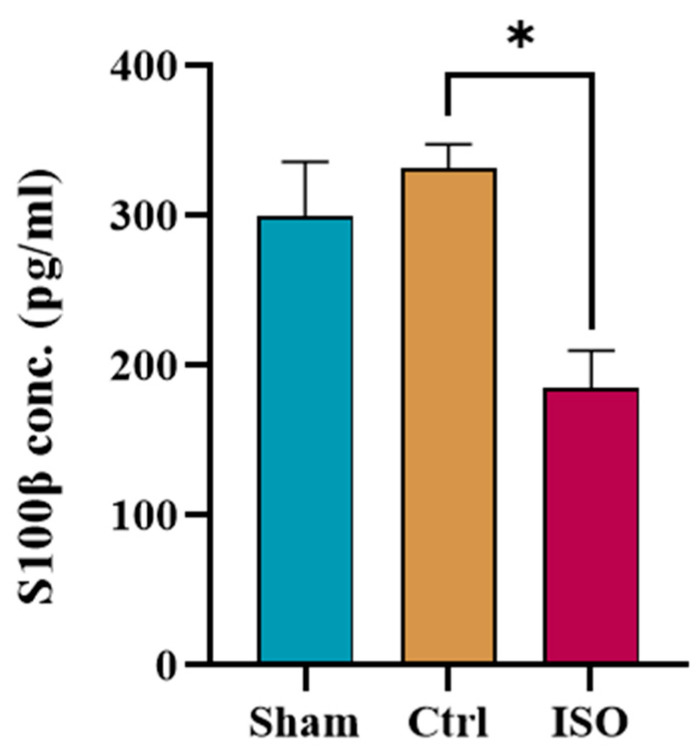
Effect of ISO on S100β protein levels. The ISO-treated group showed a significant (* *p* = 0.021) decrease in S100β protein expression compared to the Ctrl group. Ctrl = Control, ISO = Isorhamnetin.

S100β levels were downregulated by ISO treatment. A statistically significant difference (* *p* = 0.021) in S100β expression levels was observed in the ISO-treated group as compared to the Ctrl and Sham groups (Figure 7).

### 2.8. ISO Modulates the Expression of Inflammatory Markers

**Figure 8:**
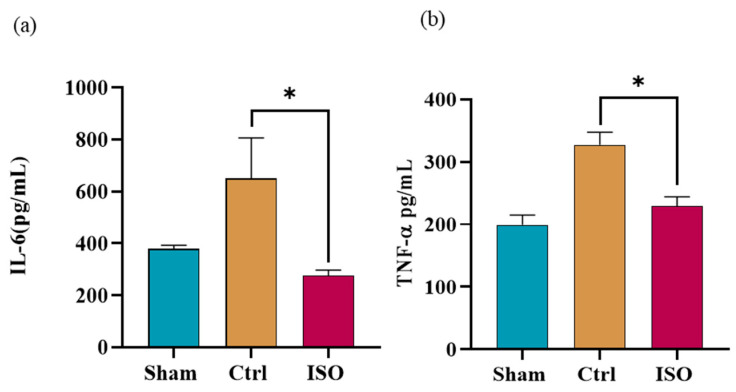
Effect of ISO treatment on inflammatory markers. Multiple-comparison tests for inflammatory biomarkers indicated potential anti-inflammatory effects of ISO. (**a**) The ISO-treated group, in contrast to the Ctrl group, showed a statistically significant (* *p* = 0.049) decrease in IL-6 expression. (**b**) TNF-α expression showed a significant decline (* *p* = 0.004) in the ISO-treated group compared with the Ctrl group. Control = Ctrl, ISO = Isorhamnetin.

Proinflammatory cytokines were modulated by ISO treatment. A statistically significant difference (* *p* = 0.049) in IL-6 expression was observed between ISO-treated and Ctrl groups. A statistically significant (* *p* = 0.004) difference in TNF-α expression between ISO-treated and Ctrl groups was observed (Figure 8a,b).

### 2.9. Impact of ISO Treatment on Hb, WBC, and Platelet Count

**Figure 9:**
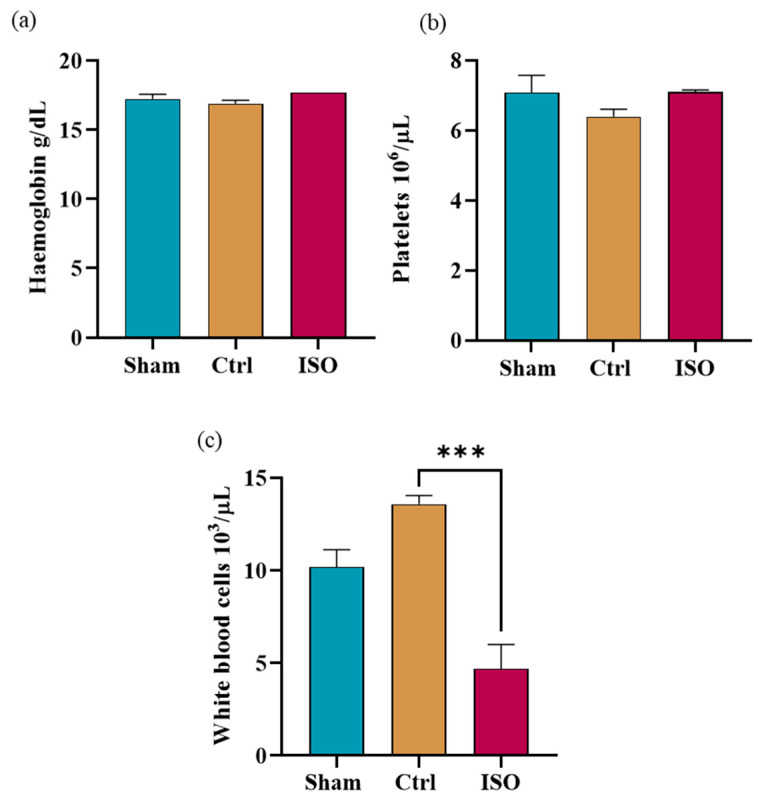
Effect of ISO treatment on Hb, WBCs, and platelet count. (**a**) Hb levels did not differ significantly between the ISO-treated and Ctrl groups (*p* = 0.258). (**b**) The ISO-treated group showed a non-significant increase in platelet counts (*p* = 0.273) when compared to the Ctrl group. (**c**) WBC count decreased significantly in the ISO-treated group (*** *p* < 0.001) in comparison to the Ctrl group.

Hb levels showed a statistically non-significant difference (*p* = 0.258) (Figure 9a), platelet count presented a non-significant difference (*p* = 0.273) (Figure 9b), and WBC count showed a significant difference among all groups (*** *p* < 0.001) (Figure 9c).

### 2.10. Impact of ISO Treatment on Differential Count

**Figure 10:**
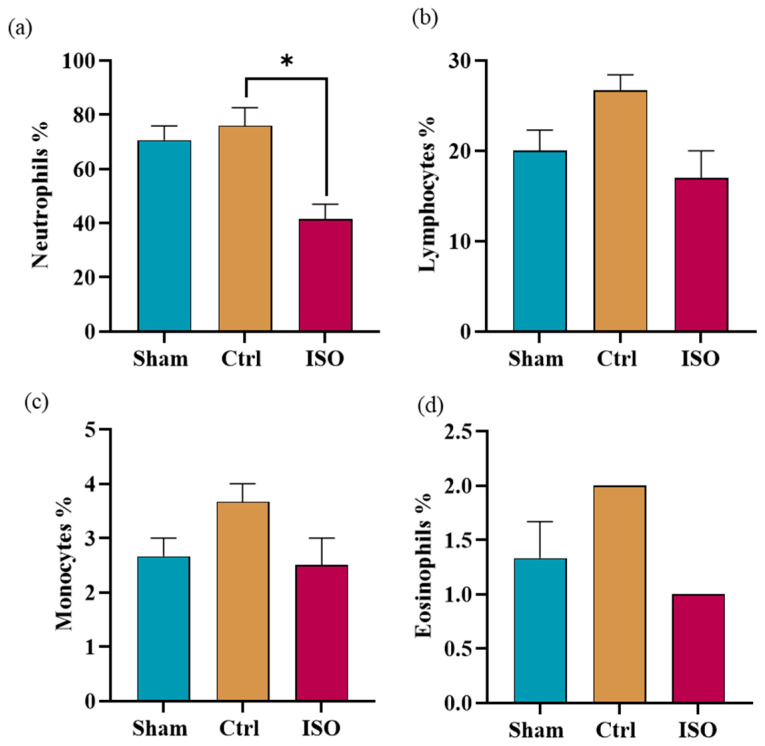
Effect of ISO treatment on differential count. Multiple comparisons of one-way ANOVA showed (**a**) a significant (* *p* = 0.028) decrease in neutrophil count. (**b**), (**c**) and (**d**) showed a non-significant (*p* = 0.073), (*p* = 0.142), (*p* = 0.064) decrease in the count of lymphocytes, monocytes, and eosinophils, respectively, in the ISO-treated group as compared to Ctrl. Ctrl = Control, ISO = Isorhamnetin.

One-way ANOVA presented a significant (* *p* = 0.028) difference in neutrophil count, as shown in Figure 10a. A statistically non-significant difference was presented by lymphocytes (*p* = 0.073), monocytes (*p* = 0.142), and eosinophils (*p* = 0.064) count among all groups (Figure 10b–d).

### 2.11. Impact of ISO on Muscle Fiber Morphology

**Figure 11:**
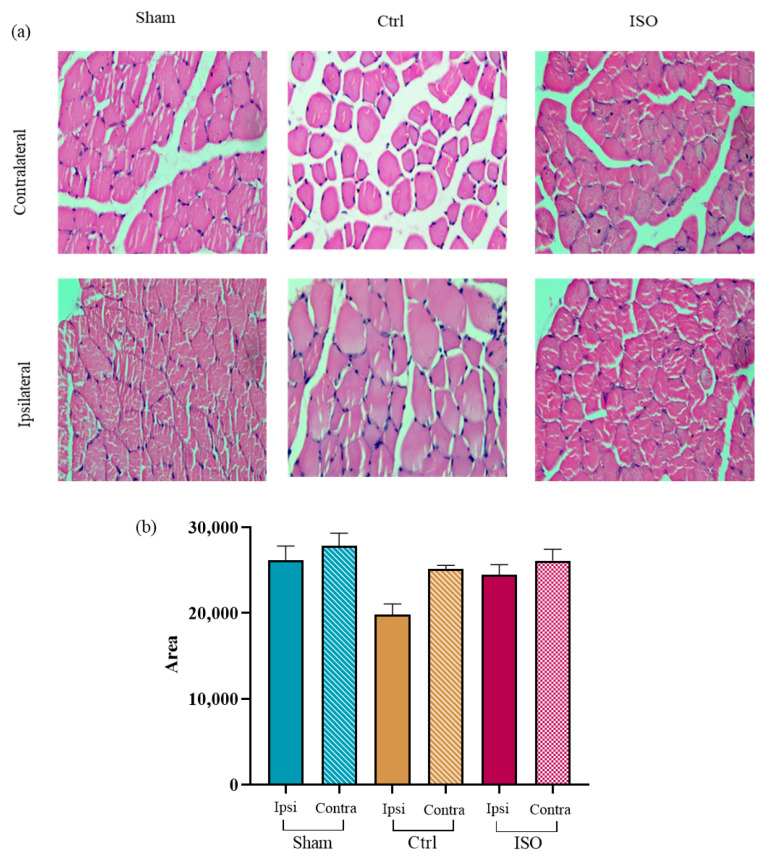
Effect of ISO treatment on the muscle fiber morphology (**a**) Representative micrographs of tibialis anterior muscle cross-sections of Sham, Ctrl, and ISO-treated groups. H&E-stained sections were examined and imaged using a compound microscope (XSZ 107BN, Hefei, China) at 40× magnification. (**b**) Muscle fiber morphology (area) showed a significant difference among the ISO-treated, Sham, and Ctrl groups (*p* = 0.013). Ctrl = Control, ISO = Isorhamnetin.

A statistically significant difference (*p* = 0.013) in muscle cross-sectional area was observed among the ISO-treated, Ctrl, and sham groups (Figure 11b).

### 2.12. Statistical Analysis

All results are shown as mean ± SEM. GraphPad Prism was used to analyze the data statistically (version 8.4.2). Tukey’s multiple comparisons test was performed after ANOVA to compare the means of all groups. A value <0.05 of *p* was considered statistically significant.

## 3. Discussion

PNI is a significant problem characterized by prolonged recovery, a poor prognosis, and complex regenerative processes. These factors not only place a significant strain on society but also pose psychological challenges for the affected ones, their family, and even the whole society [30]. As a result, patients with nerve injuries need additional strategies to aid in their functional recovery. Due to the unavailability of any gold standard and effective therapy, the functional restoration of affected people always remains an unmet challenge. There is a dire need to explore and evaluate new drug candidates to address this issue. Here comes a ray of hope: natural compounds can play a pivotal role in developing a new therapeutic approach to accelerate nerve regeneration and restore timely normal function of the injured nerve. Unlike the central nervous system, Schwann cells in the peripheral nervous system play a crucial role in several nerve-healing processes, including axonal growth, remyelination, and regeneration [31]. Therefore, targeting Schwann cells can demonstrate encouraging results.

Natural compounds have been widely used since ancient times for their antioxidant, anti-inflammatory, analgesic, antidiabetic, and antimicrobial properties [32,33]. Here, we evaluated ISO (3-methylquercetin), a common O-methylated flavonol found in medicinal herbs and plants of the Polygonaceae family [34], for its possible effect on muscle function rehabilitation following an injury to the sciatic nerve using a rodent model.

There is currently no study evaluating the possible role of ISO in peripheral nerve lesions. This investigation examines the potential impact of ISO on improving functional recovery. Concurrent daily measurements of body mass and food intake did not manifest a statistically significant difference. They supported the fact that the animal feeding habits in the current study were unaffected by treatment.

The ISO treatment showed improvement in motor function recovery at day 6, which became noticeable on day 11, as assessed by SFI and muscular grip strength. We know that the muscle gripping force is an indication of the number of motor units reinnervated. Therefore, it could be assumed that neuromuscular junctions were restored as a result of treatment [35]. In addition to motor function recovery, we also examined sensory function recovery, as the sciatic nerve is a mixed nerve. Results revealed that the ISO-treated group showed a gradual restoration of sensory responses, noticeable even on day 5. These findings demonstrate that ISO exerts neuroprotective effects and increases the sensory threshold following injury to the sciatic nerve.

The findings revealed increased blood glucose levels in the Ctrl group after injury, consistent with several studies showing hyperinsulinemia due to peripheral tissues lacking insulin-sensitizing properties, leading to hyperglycemia [36]. The ISO treatment effectively restored blood glucose levels to normal, non-diabetic levels [25].

Oxidative stress is a major contributor to post-injury damage, which also impedes the recovery of peripheral nerve function. Flavonoid molecules are advantageous in preventing chronic diseases due to their potent anti-inflammatory and antioxidant properties [26]. ISO showed protection against oxidative stress in human Retinal Pigment Epithelial cells [37]. Antioxidants primarily protect against the harmful effects of free radicals. The total antioxidant capacity of a biological sample is the assessment of its antioxidant state and measures its response to free radical species [38]. Reports indicate that ISO significantly reduced total oxidative stress and increased total antioxidant capacity, supporting its antioxidant potential. Although Reactive oxygen species (ROS) contribute to normal cellular functions in peripheral nerve tissue, most studies have emphasized their detrimental effects when present in excess, leading to oxidative stress [10].

ROS trigger the release of MDA, a highly reactive byproduct that interacts with proteins and nucleic acids, causing damage to various tissues and cells [39]. Our findings indicate that ISO did not significantly decrease plasma MDA levels, suggesting that it could serve as an effective antioxidant by reducing lipid peroxidation or enhancing free radical scavenging. Catalase (CAT), a heme-containing tetrameric enzyme, is a common antioxidant produced naturally by the body when exposed to oxygen. CAT protects cells by detoxifying H_2_O_2_ and plays a key role in developing tolerance to oxidative stress as an adaptive response [40]. The ISO-treated group showed a trend toward increased catalase activity.

S100β protein, a member of the S100 protein family, is a homodimeric acidic protein. Astrocytes primarily express S100B and have both autocrine and paracrine effects on glial and neuronal cells [41]. S-100b protein is restricted in the cytoplasm and membranes of Schwann cells in both myelinated and non-myelinated fibers in sciatic nerves, according to earlier research based on immunocytochemical data [42]. S100β protein contributes to the regenerative process. Therefore, after nerve injury, serum S100β levels increased and decreased with functional recovery. The ISO-treated group showed a significant decrease in S100β expression. This might be related to the enhancement of endogenous neurogenesis after ISO treatment. Cytokines are the primary regulators of the inflammatory response, triggering an acute phase response to shield the body from irritation, damage, and infection. It is crucial to keep in mind that chronic inflammation might result from an overabundance of proinflammatory reactions [43]. The ISO significantly decreased IL-6 and TNF-α expression. This anti-inflammatory mechanism of isorhamnetin appears to be mediated through the modulation of multiple inflammatory mediators [44].

Because platelets and other white blood cells have numerous roles in supporting axonal regeneration in the nervous system, their numbers were assessed [45]. Erythropoietin also promotes red blood cell production and accelerates motor function recovery in the sciatic nerve injury model [46]. The haematological outcomes of the Ctrl, sham, and ISO-treated groups were relatively inconsistent. We found that the ISO-treated group had non-significantly higher Hb levels. This raises the possibility that functional recovery is due to elevated erythropoietin levels induced by treatment [47]. The ISO-treated group had significantly higher WBC counts. Lower WBC counts, especially neutrophils, in the ISO-treated group may be linked to ISO’s anti-infectious properties. Infections can occur due to sensory and functional loss following injury and can be reversed with treatment.

If a nerve is injured, electrical impulses cannot reach the target muscle. Muscle fibers atrophy when these signals are absent for an extended period. The tiny cross-sectional area and irregular shape of muscle fibers often reflect their health [48]. A significant improvement in the cross-sectional area of muscle fibers was observed in response to ISO treatment.

Nevertheless, NF-κB was not directly evaluated in the present study; the observed modulation of S100β and proinflammatory cytokines suggests a potential involvement of NF-κB–mediated inflammatory signalling in ISO-induced neuroregeneration, warranting further investigation. The present study primarily relied on functional assessments of nerve regeneration, including SFI, grip strength, and sensory testing; however, incorporating structural and molecular evaluations, such as transmission electron microscopy and immunohistochemistry, would further strengthen and substantiate the observed regenerative effects. However, future studies using neuronal or Schwann cell models are needed to further clarify the neuroregenerative potential of ISO at the cellular level.

## 4. Materials and Methods

### 4.1. Compound

**Figure 12:**
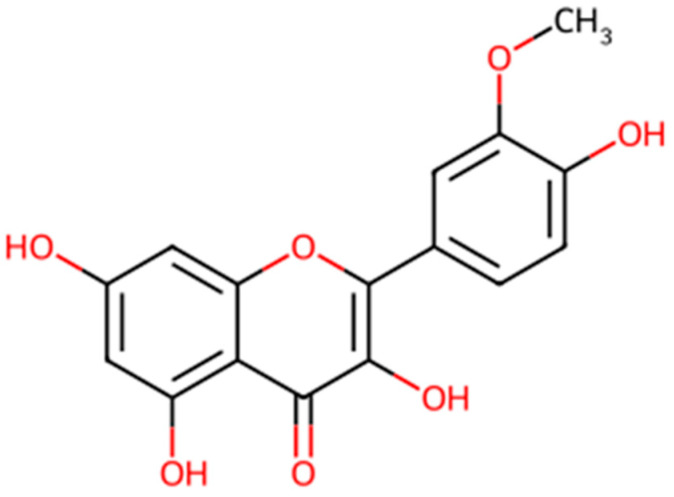
Chemical structure of isorhamnetin.

The ISO was purchased from Nanjing Forever, Nanjing, China (LOT# N63259874). The chemical structure of the compound “https://www.chemspider.com/Chemical-Structure.4444973.html”(accessed on 24 July 2025) is shown in Figure 12.

### 4.2. MTT Assay

We first determined the effects of ISO on the cell viability and mitochondrial functions using HepG2 cells in an MTT assay. To determine whether ISO treatment affects HepG2 cell survival, cells were treated with 25, 50, and 100 µg/mL ISO. The cells were grown at a density of 2 × 10^4^ cells per well. The plate was kept in a CO_2_ incubator for twenty-four hours. Fresh Dulbecco’s Modified Eagle Medium (DMEM) containing 10 µL of the tested substance was used in place of the culture medium. The cells were incubated for another 48 h. The live cells reduced the 5 mg/mL MTT solution into purple formazan crystals, which were pipetted into each well after incubation. Each well was filled with 150 µL of dimethyl sulfoxide (DMSO). At 560 nm, the optical density was measured [49].

Percent cell death = [(OD of negative control − OD of sample)/OD of negative control] × 100.

### 4.3. Experimental Animals

Healthy male albino mice (BALB/c) weighing between 25 and 35 g and with an average age of 8 to 10 weeks were acquired from the Department of Physiology’s animal housing facility, Government College University, Faisalabad. Every animal was kept in a single cage (plastic rodent cage). The housing specifications, which include a room temperature of 23 to 27 °C, a 12-h light and 12-h dark cycle, optimal humidity, and an unlimited supply of food and water, were maintained throughout the experiment and a one-week acclimatization phase. All analyses were carried out during the light cycle. ISO was prepared as a stock solution and diluted in DMSO to a final concentration of 10 mg/kg [25].

### 4.4. Surgery

Following a week of acclimatization, the experimental mice were surgically prepared to induce a nerve injury. They received deep anaesthesia, which involved intraperitoneal injections of a combination of ketamine and xylazine at a dose of 70 mg/kg and 5 mg/kg, respectively. Following a smooth shave of the surgical location, which was done on the mid-thigh region of the hind limbs of the animals, a tiny incision was made to reveal the sciatic nerve in the mid-thigh region of the animals. Each mouse’s nerve was crushed for 15 s using the identical set of forceps and consistent pressure. The presence of a translucent ring would confirm that the nerve had been crushed. To prevent infection, pyodine was applied to the incision after the skin was sutured with two to four stitches [50].

### 4.5. In Vivo Experimental Design

Thirty mice were randomly assigned to three groups (*n* = 10 per group): (1) Ctrl group: DMSO and normal saline were administered intraperitoneally in equal volumes after injury; (2) Sham group: DMSO and normal saline were administered intraperitoneally in equal volumes after sciatic nerve exposure; and (3) ISO-treated group: ISO (10 mg/kg) dissolved in DMSO were administered intraperitoneally after injury. All groups were fed regular chow and provided with sufficient water throughout the experiment. The mice in the Ctrl and ISO-treated groups were operated on, whilst the animals in the sham group had their sciatic nerves exposed but left intact without crushing.

### 4.6. Physical Parameters

#### Weight and Food Consumption

The daily body weight and food intake of each experimental mouse were recorded throughout the experiment. The difference between the weight of the offered and the leftover diet in the cage on each day was estimated to record the amount of food consumed [51].

### 4.7. Behavioral Analyses

#### 4.7.1. Sciatic Functional Index

The sciatic functional index (SFI) was used to evaluate motor functional recovery. To measure SFI, walking track analysis was performed. The hind paws were painted with ink, and the mouse was allowed to walk on a 50 cm-long, narrow wooden track with white paper on the floor. The representative footprint was selected to measure the parameters used to calculate the SFI. Between the second and fourth toes, the distance is known as the intermediate toe spread (IT). Print length (PL) is the distance between the heel and the tip of the third toe, whereas toe spread (TS) is the distance between the first and fifth toes. NPL, NTS, and NIT represent the normal group’s readings, whereas the Experimental group’s readings are represented by EPL, ETS, and EIT [52].

SFI was calculated by using this formula [52].

$$SFI=\left(-38.3\times \frac{EPL-NPL}{NPL}\right)+(109.5\times \frac{ETS-NTS}{NTS})+(13.3\times \frac{EIT-NIT}{NIT})-8.8$$

#### 4.7.2. Muscle Grip Strength

The most commonly used method for evaluating motor function recovery after sciatic nerve injury is to use a grip strength meter to measure hind-limb gripping force. It enables the in vivo calculation of muscle strength by allowing the mouse to hold a metal bar of a grip strength meter (Bioseb, Chaville, France) that displays the gripping force in Newton (N) on the screen. Hind paw grip strength was quantified bilaterally (ipsilateral and contralateral to the injury site), and comparative analyses between Ctrl and treatment groups were performed to evaluate functional recovery [32].

#### 4.7.3. Hot Plate Test

The hot plate test was used to assess the sensory functional recovery. The mouse was allowed to spend one minute with the non-working hot plate (SCILOGEX MS7-H550-S LED digital 7×7 Hot-plate stirrer, SCILOGEX, Rocky Hill, CT, USA) before the actual testing. Following that, they were allowed to remain in contact with the apparatus, set at 56 ± 2 °C, with their operated hind paw until they responded. This duration was recorded as the hot plate latency (HPL). The latency period was measured using a stopwatch and defined as the withdrawal reflex latency (WRL). If the mice did not respond after 30 s, they were removed, and the latency was recorded as 30 s. For each mouse, three readings were recorded, each separated by 2 min [48].

#### 4.7.4. Pinprick Test

The pinprick test is a method for evaluating recovery of sensory function following nerve injury. The lateral portion of the hind paw’s plantar surface was hypothetically separated into five sections while the mouse was housed in a wire mesh cage. Each region was gently pinpricked with a size “000” Austerlitz insect pin, and a quick paw removal was noted as a positive reaction. The experimental paw was tested from the heel (A) to the furthest lateral toe (E). A “1” was assigned to the region for a good response, and a “0” for no response [53]. The mouse exhibiting a positive reaction on each of the five locations was thus assigned a score of 5, indicating a completely developed sensory response. A score of less than five indicates partial recovery, whereas a score of zero indicates total functional loss [50].

### 4.8. Measurement of Random Blood Glucose

To determine the effect of glucose on exacerbating neurotic events at the site of nerve damage, glycemic levels were monitored both before and after nerve injury induction. As described in earlier research, a glucometer (Accu-Chek) was used to measure the glycemic level in each group by placing a drop of blood from the mouse’s tail on a glucometer strip [50,54].

### 4.9. Biochemical Analyses

All animals were sacrificed at the end of the experiment, their blood was drawn, centrifuged, and serum was separated and preserved for biochemical assays.

### 4.10. Oxidative Stress Markers

#### 4.10.1. Total Antioxidant Capacity

A biosystem’s antioxidant capacity is its ability to scavenge free radicals and the related malfunctions. TAC in the serum samples was assessed. In this assay, ABTS (2,2′-azino-bis(3-ethylbenzothiazoline-6-sulfonic acid)) was oxidized by hydrogen peroxide under acidic conditions, producing a chromogenic radical cation; the resulting ABTS solution was green. The addition of serum samples to this medium resulted in bleaching, the extent of which depended on the presence or absence of antioxidants. The absorbance of the mixture was measured using an automated chemistry analyzer (BIOLAB310) at 650 nm. The analysis was calibrated using vitamin C. The TAC units were given in mmol of Vit C Eq/L [55].

#### 4.10.2. Total Oxidant Status

Total oxidant stress is the body’s overall oxidative condition [56]. The serum was tested for total oxidant status (TOS) to assess oxidative stress in each group after sciatic nerve injury. Following a spectrophotometer (BIOLAB 310), the total oxidants present in the serum sample were determined. The dianisidine–ferrous ion combination serves as the substrate in this assay. In an acidic solution, xylenol orange and the ferric ion combined to generate a colourful complex. The concentration of oxidants in this complex was directly correlated with colour intensity, reflecting their ability to oxidize ferrous ions to ferric ions. The absorbance of this colourful mixture was measured at 560 nm using an automated analyzer (BIOLAB 310). The analysis was calibrated using hydrogen peroxide. Results were reported as µmol of H_2_O_2_ equivalent after calibration with H_2_O_2_ [57].

### 4.11. Measurement of MDA Level

MDA (malondialdehyde) generation was measured using the Thiobarbituric Acid Reactive Substances (TBARS) assay to evaluate lipid peroxidation [58]. 100 µL of the sample was added to 900 µL of distilled water, then 500 µL of TBA was added. After cooling, the mixture was centrifuged at 4000 rpm for 10 min. After removing the supernatant, analysis of malondialdehyde (MDA) was done using spectrophotometry (UH5300; HITACHI, Tokyo, Japan), as previously described in [59].

### 4.12. Evaluation of Catalase Activity

This test was used to evaluate the body’s capacity to counteract oxidative damage. First, an Eppendorf tube (Hamburg, Germany) was filled with 500 µL of distilled water, then 50 µL of the sample was added. After incubation at 37 °C for 3 min, 50 µL H_2_O_2_ was added to the mixture, followed by 500 µL of dichromate. After incubation in a water bath for 10 min, the samples were allowed to equilibrate to room temperature. The samples were centrifuged for five minutes at 25,000 rpm. At a wavelength of 545 nm, measurements were made using a BIOLAB 310 chemical analyzer (URIT, Guilin, China) [60].

### 4.13. Evaluation of Serum S100β Level

An ELISA kit (Glory Science, Shanghai, China, LOT: 202505) was used to measure S100β in serum samples from ISO-treated mice. 10 µL of the test samples were added to each well of the microtiter plate after 50 µL of the standards were added to the corresponding well, then 40 µL of sample diluent was added. Subsequently, 100 µL of HRP-conjugate reagent was dispensed into each well, after which the plate was sealed with an adhesive strip and incubated at 37 °C for 60 min. Following incubation, the wells were aspirated and subjected to five consecutive washes with the washing solution. Each well was supplemented with 50 µL of chromogen solution A, followed by 50 µL of chromogen solution B. After 15 min of incubation at 37 °C, 50 µL of stop solution was dispensed into each well of the plate. The wells turned yellow. A microplate reader (URIT-660, Guilin, China) was used to measure the optical density (OD) at 450 nm in 15 min.

### 4.14. Evaluation of Inflammatory Biomarkers

ELISA kits (BT LAB, Shanghai, China) were used to measure proinflammatory cytokines, including interleukin-6 (IL-6) and tumour necrosis factor-α (TNFα), in mouse serum. For IL-6 Cat. No. E0135Ra was used for TNFα, Cat. No. E00764Ra was used according to the manufacturer’s procedure. Sample wells were filled with 40 µL of sample and 10 µL of anti-IL-6 antibody. The sample and reference wells were then filled with 50 µL of streptavidin-HRP. The plate was then incubated at 37 °C for 60 min. After that, the plate was cleaned five times for 30 to 1 min each using 300 µL of buffer wash. Subsequently, 50 µL of each substrate solution (A and B) was dispensed into each well and incubated under controlled conditions to allow colour development. The enzymatic reaction was then terminated by adding 50 µL of stop solution to each well. The hue blue instantly became yellow. Within 10 min of applying the stop solution, the optical density of each sample was measured using a microplate reader set to 450 nm. Each sample was examined in triplicate (*n* = 3). An identical procedure was carried out for the quantification of TNF-α using its corresponding ELISA kit.

### 4.15. Quantitative Analysis of Blood Cell Composition

A blood sample was obtained after the decapitation. Blood was stored in EDTA-anticoagulated whole blood sample vials, and a haematology analyzer (Swelab AlfaLyse, Stockholm, Sweden) was used to determine haemoglobin concentration, number of white blood cells, neutrophils, eosinophils, lymphocytes, monocytes, and platelet count. Platelets were counted according to the Brecher–Cronkite method [61].

### 4.16. Histology

Following dissection, the tibialis anterior muscles from both hind limbs were excised and fixed in 10% neutral-buffered formalin (pH 6.8) for 24 to 48 h. After being left to wash overnight to get rid of the fixative, the tissue was further dehydrated in a succession of alcohols. A Leica RM2125 microtome (LEICA, Deer Park, IL, USA) was used to cut a 5 µm piece of tissue that had been fixed in paraffin. Hematoxylin and eosin (H&E) stains were applied to sections that had been placed on slides. The morphometric pattern of the ipsilateral and contralateral muscles was seen and assessed at 40× using a compound microscope (XSZ 107BN, Hefei, China). Lastly, a camera (Optika B1, Ponteranica, Italy) was used to capture pictures. Afterward, ImageJ, version 1.52, was used to quantify the cross-sectional area of each muscle fiber within an evenly selected region of the image. The average of each fiber in each image was calculated and compared across all groups [32,62].

## 5. Conclusions

Based on the results, we demonstrate that ISO exerts significant neuroprotective effects against sciatic nerve injury in a mouse model, as evidenced by enhanced functional recovery, reduced oxidative stress and inflammation, and maintenance of muscle fibre morphology, underscoring its promise as a treatment for peripheral nerve restoration. Its graded-dose cytotoxicity also suggests its potential safety, suggesting that ISO might be a good option for therapeutic use in neuroprotection and neuroregeneration. Further investigation is warranted to elucidate the molecular mechanisms by which this compound facilitates accelerated axonal regeneration.

## Data Availability

The original contributions presented in this study are included in the article. Further inquiries can be directed to the corresponding author.

## Abbreviations

The following abbreviations are used in this manuscript:

PNI	Peripheral nerve injury
ISO	Isorhamnetin
Ctrl	Control
i.p	Intraperitoneal
DMEM	Dulbecco’s modified Eagle media
DMSO	Dimethyl sulfoxide
SFI	Sciatic functional index
IT	Intermediate toe spread
PL	Print length
TS	Toe spread
HPL	Hot plate latency
WRL	Withdrawal reflex latency
ABTS	(2,2′-azino-bis(3-ethylbenzothiazoline-6-sulfonic acid))
MDA	Malondialdehyde
